# Adapted time-varying covariates Cox model for predicting future cirrhosis development performs well in a large hepatitis C cohort

**DOI:** 10.1186/s12911-021-01711-7

**Published:** 2021-12-14

**Authors:** Lauren A. Beste, Xuefei Zhang, Grace L. Su, Tony Van, George N. Ioannou, Brandon Oselio, Monica Tincopa, Boang Liu, Amit G. Singal, Ji Zhu, Akbar K. Waljee

**Affiliations:** 1 General Medicine Service, Veterans Affairs Puget Sound Healthcare System, Seattle, WA USA; 2 Department of Medicine, Veterans Affairs Puget Sound Healthcare System, Seattle, WA USA; 3 Department of Statistics and Biostatistics, University of Michigan, Ann Arbor, MI USA; 4 Michigan Integrated Center for Health Analytics and Medical Prediction (MiCHAMP), Ann Arbor, MI USA; 5 Gastroenterology Service, VA Ann Arbor Healthcare System, 2215 Fuller Road, Gastroenterology 111D, Ann Arbor, MI 48105 USA; 6 Department of Internal Medicine, Michigan Medicine, Ann Arbor, MI USA; 7 Center for Clinical Management Research, VA Ann Arbor Healthcare System, Ann Arbor, MI USA; 8 Institute for Healthcare Policy and Innovation, University of Michigan, Ann Arbor, MI USA; 9 Gastroenterology Service, Veterans Affairs Puget Sound Healthcare System, Seattle, WA USA; 10 Department of Medicine, University of Washington, Seattle, WA USA; 11 Harold C. Simmons Comprehensive Cancer Center UT Southwestern Medical Center, Dallas, TX USA; 12 Division of Digestive and Liver Diseases, Department of Internal Medicine, UT Southwestern Medical Center, Dallas, TX USA; 13 Department of Internal Medicine, Parkland Health and Hospital System, Dallas, TX USA

**Keywords:** Veterans, Sustained virologic response, Hepatitis C virus, Survival model, Prediction

## Abstract

**Background:**

Patients with hepatitis C virus (HCV) frequently remain at risk for cirrhosis after sustained virologic response (SVR). Existing cirrhosis predictive models for HCV do not account for dynamic antiviral treatment status and are limited by fixed laboratory covariates and short follow up time. Advanced fibrosis assessment modalities, such as transient elastography, remain inaccessible in many settings. Improved cirrhosis predictive models are needed.

**Methods:**

We developed a laboratory-based model to predict progression of liver disease after SVR. This prediction model used a time-varying covariates Cox model adapted to utilize longitudinal laboratory data and to account for antiretroviral treatment. Individuals were included if they had a history of detectable HCV RNA and at least 2 AST-to-platelet ratio index (APRI) scores available in the national Veterans Health Administration from 2000 to 2015, Observation time extended through January 2019. We excluded individuals with preexisting cirrhosis. Covariates included baseline patient characteristics and 16 time-varying laboratory predictors. SVR, defined as permanently undetectable HCV RNA after antiviral treatment, was modeled as a step function of time. Cirrhosis development was defined as two consecutive APRI scores > 2. We predicted cirrhosis development at 1-, 3-, and 5-years follow-up.

**Results:**

In a national sample of HCV patients (n = 182,772) with a mean follow-up of 6.32 years, 42% (n = 76,854) achieved SVR before 2016 and 16.2% (n = 29,566) subsequently developed cirrhosis. The model demonstrated good discrimination for predicting cirrhosis across all combinations of laboratory data windows and cirrhosis prediction intervals. AUROCs ranged from 0.781 to 0.815, with moderate sensitivity 0.703–0.749 and specificity 0.723–0.767.

**Conclusion:**

A novel adaptation of time-varying covariates Cox modeling technique using longitudinal laboratory values and dynamic antiviral treatment status accurately predicts cirrhosis development at 1-, 3-, and 5-years among patients with HCV, with and without SVR. It improves upon earlier cirrhosis predictive models and has many potential population-based applications, especially in settings without transient elastography available.

**Supplementary Information:**

The online version contains supplementary material available at 10.1186/s12911-021-01711-7.

## Background

Approximately 20–25% of patients with untreated hepatitis C virus (HCV) infection will progress to cirrhosis within 30 years. Though individual rates of progression vary depending on comorbidities and other risk factors [1], modern direct acting antiviral (DAA) medications have outstanding efficacy and can eradicate HCV in nearly all cases [2]. Viral eradication clearly reduces progression to cirrhosis and lowers the risk of mortality [3–6]. However, the rate of hepatic fibrosis regression varies between individuals. In some, liver disease may continue progressing even after successful antiviral treatment, particularly given the emergence of non-alcoholic fatty liver disease [7].

Hepatic fibrosis and necroinflammation are powerful predictors of future disease progression [8]. Liver biopsy, historically the criterion standard for assessment of hepatic fibrosis, is invasive, costly, and associated with complications, making it impractical for routine monitoring of all patients with HCV [9]. Transient elastography, while promising, is not universally available and may not be obtainable in low-resource settings [10]. Many cross-sectional studies have tried to use laboratory data or other non-invasive methods to stage hepatic fibrosis in individuals with HCV at a single time point, yet few have aimed to predict future liver disease progression and none have incorporated dynamic antiviral treatment status into predictive models [11]. As a result, available cirrhosis prediction models have unknown generalizability to the expanding group who achieve sustained virologic response (SVR) after HCV antiviral therapy. No available laboratory-based models accurately predict the risk of progression to cirrhosis after SVR, with the result that life-threatening liver disease complications, such as hepatocellular carcinoma or esophageal varices, could develop and progress undetected after antiviral treatment.

The few cirrhosis predictive models that do exist have methodologic limitations and only achieve marginal discrimination in predicting fibrosis progression [12–14]. Most importantly, earlier models using traditional regression-based methods reduced laboratory data to a single value (e.g., baseline value, mean, maximum, minimum, etc.) [12–15]. This approach obscures the trajectory of key laboratory data, often an important clue to the ongoing development of cirrhosis [14]. We sought to develop an improved cirrhosis prediction model using survival analysis, an ideal technique given the variable length of time until development of cirrhosis, while also incorporating the full spectrum of laboratory data and HCV treatment status.

## Methods

### Study population and data collection

We obtained data from the VHA Corporate Data Warehouse, a continually updated electronic repository of demographic, laboratory, pharmaceutical, and other clinical data for Veterans under VHA care. We identified all patients in the VHA system with a history of HCV, defined as the lifetime presence of at least one positive HCV RNA test from January 1, 2000, to January 1, 2016 (n = 280,494). We defined HCV treatment as the receipt of at least one dose of an antiviral medication approved by the US Food and Drug Administration for the treatment of HCV on or before December 31, 2015. SVR was defined as of December 31, 2016, as the permanent absence of detectable HCV RNA after antiviral treatment. Patients were followed for the development of cirrhosis or death through January 1, 2019. We required patients to have at least two AST-to-platelet ratio index (APRI) scores (n = 231,566). APRI is a widely used non-invasive method for assessing fibrosis stage among patients with HCV, with excellent accuracy in detecting advanced fibrosis and cirrhosis. We defined APRI using the standard formula APRI = 100*(AST (U/L)/40)/platelet count (1000/µL) [16]. Component laboratory values were required to be drawn within 30 days of one another and could occur in inpatient or outpatient settings.

**Fig. 1:**
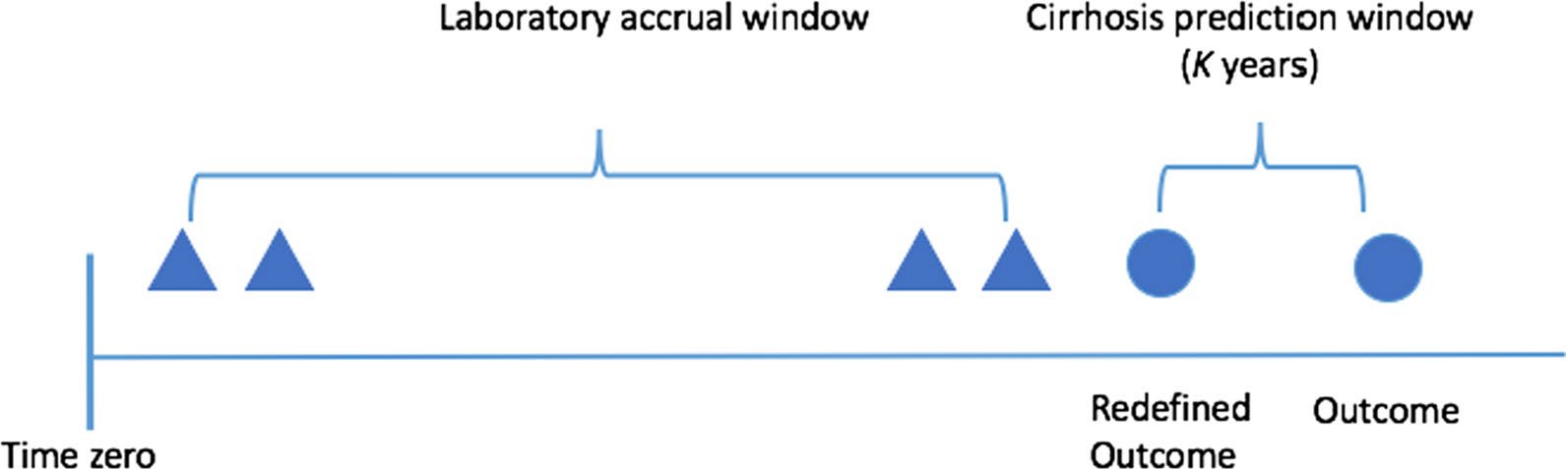
Adapted time-varying covariates model design

**Fig. 2:**
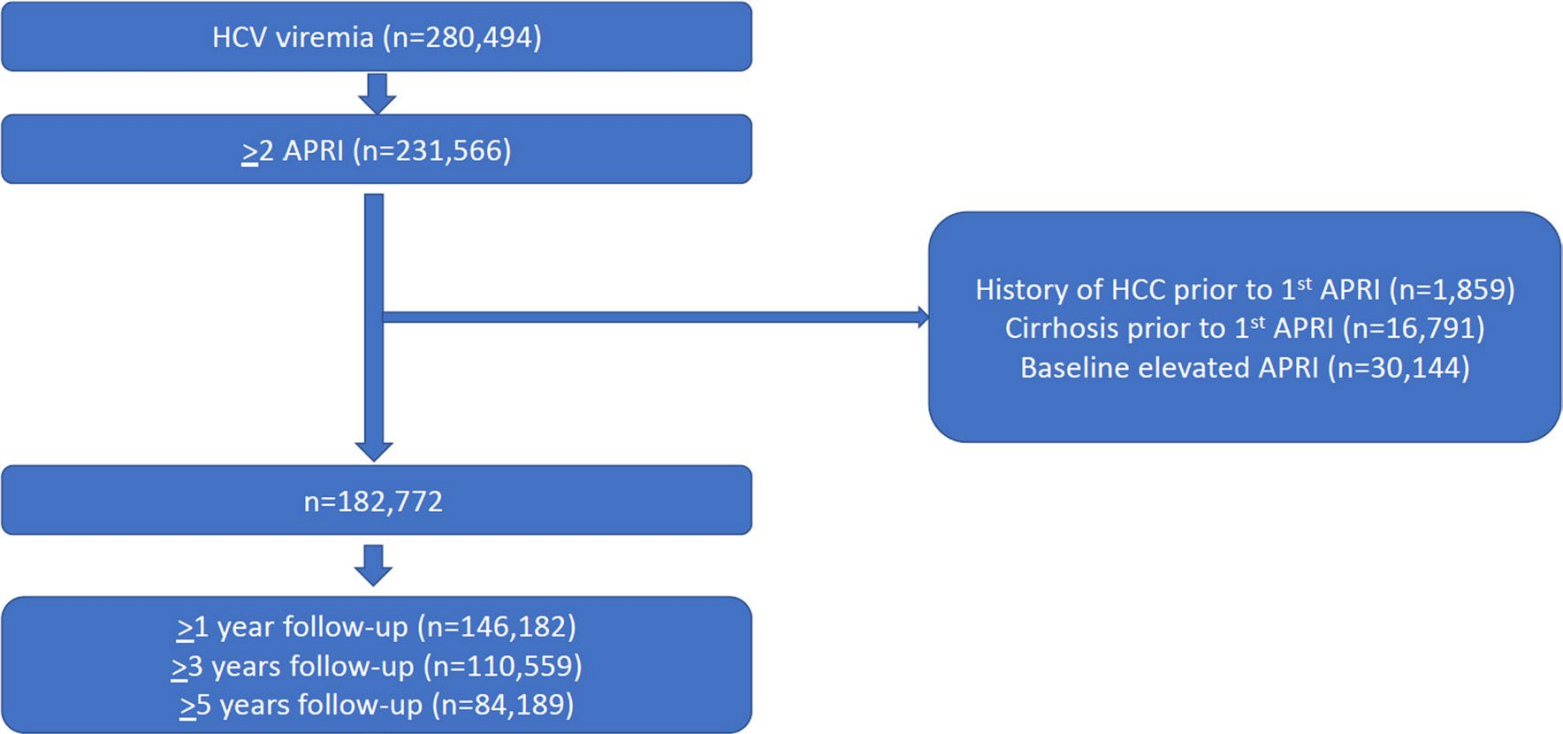
Cohort development

Cohort entry was defined as the date of the first APRI. Time-zero was defined as the time of entry into the cohort (Fig. 1). We excluded patients with known or suspected cirrhosis at baseline or a history of hepatocellular carcinoma, defined by relevant International Classifications of Diseases (ICD) codes prior to or within 1-year after cohort entry (n = 18,650) (Additional file 1: Table S1) or baseline APRI > 2.0 (n = 30,144) [16]. Our final cohort contained 182,772 patients with both HCV and at least two APRI scores who were classified as non-cirrhotic at baseline (Fig. 2). The study was reviewed by the Institutional Review Board of the Ann Arbor VA Healthcare Systems and was granted a waiver of informed consent.

### Predictor variables

Predictors of interest were selected a priori based on our prior work, biological plausibility, and expert clinician opinion [13–15]. Demographic variables included age at cohort entry, sex, race, and Hispanic ethnicity. SVR was modeled as a step function of time whereby the variable value remained 0 until antiviral treatment, at which point it became 1. Laboratory predictors included aspartate aminotransferase (AST), alanine aminotransferase (ALT), AST/ALT ratio, albumin, total bilirubin, creatinine, blood urea nitrogen, glucose, hemoglobin, platelet count, white blood cell count, sodium, potassium, and chloride. INR and total protein were excluded due to a large baseline degree of missingness (50% missing and 17% missing at baseline, respectively). We used all available laboratory data points for each patient. We modeled each longitudinal laboratory predictor using a stepwise function where the value between two consecutive time points was imputed by the lab value measured at the previous time point. Specifically, if we did not have a lab measured at time-zero, we imputed the missing value with the median of the variable of all measured values of all patients at time-zero. After time-zero, any lab values missing during the accrual window (2- or 4-year window) were imputed by the closest last measured value prior to the missing value.

We considered including additional comorbidities such as alcohol use and diabetes in the model. However, in prior work we found that these additional characteristics did not significantly contribute to prediction of cirrhosis after accounting for longitudinal laboratory results (e.g., AST, glucose) already included in our models [15]. In the same earlier study, we systematically evaluated a variety of parameters for body mass index (BMI) (e.g., most recent, minimum, maximum) and found they ranked at or near the bottom of statistical importance relative to the laboratory variables. Therefore, in the current study we report these patient characteristics but limited our models to laboratory data to enhance reproducibility across systems and avoided variables such as “alcohol use” which may be documented inconsistently and depend on the accuracy of patient reporting as well as the definitions and diagnostic criteria used [15].

### Outcome variable

We defined our primary outcome, cirrhosis development, as two consecutive APRI scores > 2, as described in previous work by our group [15]. APRI has been previously validated against liver biopsy in patients with HCV and has outstanding discrimination based on area under receiver operating curve (AUROC), with performance similar to transient elastography for detecting cirrhosis [17]. Furthermore, APRI is less sensitive to the effects of age than other non-invasive markers of fibrosis, such as the FIB-4 index, and performs at least as well as FIB-4 in predicting cirrhosis after SVR [18, 19]. The observation period for each patient started on the date of the first recorded APRI and ended with occurrence of cirrhosis or censoring due to death or loss to VHA follow-up.

### Statistical analysis

*Time-varying covariates Cox model*: In classical time-varying covariates Cox models, prediction of an outcome (or “event”) in the future is not possible because computation of the survival probability at a future time would require future knowledge of covariate values. In order to predict future cirrhosis using a time-varying covariates Cox model, we redefined the notion of an event in survival analysis. Traditionally, an event consists of the occurrence of an outcome at the current time. We changed its definition as occurrence of cirrhosis *K* (1-, 3-, 5-years) years after the accrual time, where *K* is the length of prediction window to be specified by the user. The hazard function in our time-varying covariates Cox model characterizes the conditional probability that cirrhosis will subsequently develop after *K* years of additional follow-up, given no previous occurrence of cirrhosis (Fig. 1). Parameters in the model were estimated via maximizing the partial likelihood. We fit time-varying covariates Cox model using Survival R version 3.6.1 package (R Project for Statistical Computing).

#### Model evaluation

To evaluate the discriminative performance of the model, we used the area under the receiver operating characteristics curve (AUROC) to compare the predicted probability of developing cirrhosis with each patient’s observed outcome (an AUROC of 1.0 represents perfect discrimination). We predicted cirrhosis development in 1-, 3-, and 5-years using laboratory data accrual windows of 2- and 4-years after first APRI. For example, if we had 4-years of lab data, we used the first 2-years of data in the accrual window to make a prediction in the subsequent years. Accrual windows of 2- and 4-years were used as a 2-year time period as an approximate assessment of a new patient trajectory coming into the health system and a 4-year period gives a more longitudinal view to capture long-term changes. Patients censored before 1-, 3-, or 5-years were removed since their true outcomes were not available. We evaluated each prediction setting in terms of specificity, sensitivity, positive predictive value (PPV), and negative predictive value (NPV).

#### Training and testing cohorts

We created model training and testing datasets by randomly splitting the sample into 70% and 30% subsets. The random splitting process was performed 30 times to produce a more stable evaluation and to generate confidence intervals. Under each split, the time-varying covariates Cox model was fitted on the training set and evaluated on the testing set. The AUROC measures for all outcome windows and predictor windows were averaged over 30 splits. We report the representative split of training and testing data with the AUROC closest to the average AUROC over 30 splits. The best cut-off was selected by choosing the point on the ROC curve closest to where both sensitivity and specificity equal one. Specifically, we the cut-off where (1-sensitivity) ^2 + (1-specificity) ^2 is minimized.

#### Sensitivity analysis

For prediction of cirrhosis, we used an APRI cut-off > 2 as our primary outcome to maximize specificity and PPV; however, we also performed a sensitivity analysis using APRI > 1 given variation in thresholds across prior studies.

## Results

### Cohort characteristics and incidence of outcomes

Table 1 provides summary statistics for patient characteristics and baseline laboratory measurements among individuals with HCV infection and a minimum of 1 (n = 146,182), 3 (n = 110,559), and 5 (n = 84,189) years of follow-up time. Patients were 97% male and majority (51%) white, with a mean age of 52.4 (SD 8.32) years old. Baseline APRI scores were low (mean 0.668 [0.686]), as expected for a non-cirrhotic population. Patients had a mean BMI of 27.4 (SD 5.24). More than half (50.9%) carried a diagnosis of alcohol use disorder and nearly a third carried a diagnosis of diabetes (31.7%). The majority (52%) underwent antiviral treatment between 2000 and 2015 (not including the additional patients treated after 2015). Of 95,630 who received treatment, 80.3% received a DAA and 30.5% received an interferon-based regimen (10.6% received both). Median time to HCV treatment (first diagnosis of HVC infection to the first treatment) of 6.91 years and an aggregated SVR rate of 80.3%. A total of 16.2% (n = 29,566) developed cirrhosis with a median of 4.98 years to cirrhosis development after time zero.

**Table 1:** Characteristics of patients with a minimum of 1, 3, and 5 years of follow up time

	1-year minimum (n = 146,182)	3-year minimum (n = 110,559)	5-year minimum (n = 84,189)	All (n = 182,772)
Age (years), mean (SD)	52.5 (8.28)	52.4 (8.08)	52.2 (7.75)	52.4 (8.32)
Male, n (%)	141,663 (96.9%)	107,012 (96.8%)	81,449 (96.7%)	177,270 (97.0%)
*Race, n (%)*				
Black	51,206 (35.0%)	40,445 (36.6%)	32,007 (38.0%)	63,017 (34.5%)
White	74,948 (51.3%)	56,817 (51.4%)	42,834 (50.9%)	93,240 (51.0%)
Hispanic	6646 (4.5%)	4906 (4.4%)	3639 (4.3%)	8414 (4.6%)
Other	2561 (1.8%)	1913 (1.7%)	1427 (1.7%)	3212 (1.8%)
Missing	10,821 (7.4%)	6478 (5.9%)	4282 (5.1%)	14,889 (8.1%)
BMI, mean (SD)	27.5 (5.24)	27.5 (5.23)	27.6 (5.23)	27.4 (5.24)
History of alcohol use disorder	73,961 (50.6%)	56,466 (51.1%)	43,473 (51.6%)	93,045 (50.9%)
History of substance use disorder	75,350 (51.5%)	58,339 (52.8%)	45,478 (54.0%)	94,167 (51.5%)
Diabetes mellitus	47,846 (32.7%)	37,977 (34.3%)	30,125 (35.8%)	57,889 (31.7%)
Human immunodeficiency virus	4085 (2.8%)	3373 (3.1%)	2738 (3.3%)	4863 (2.7%)
Received antiviral treatment*, n (%)	75,820 (51.9%)	61,435 (55.6%)	49,338 (58.6%)	95,630 (52.3%)
SVR, n (%)	69,475 (47.5%)	56,534 (51.1%)	45,629 (54.2%)	76,854 (42.0%)
*Baseline laboratory values, mean (SD)*				
APRI	0.648 (0.692)	0.609 (0.510)	0.585 (0.473)	0.668 (0.686)
Albumin (g/dL)	3.99 (0.447)	4.01 (0.437)	4.01 (0.430)	3.98 (0.461)
Alanine aminotransferase (U/L)	65.8 (53.6)	64.2 (52.0)	63.3 (50.7)	67.1 (55.9)
Aspartate aminotransferase (U/L)	51.5 (33.4)	49.7 (31.6)	48.6 (30.6)	52.5 (34.3)
Total bilirubin (mg/dL)	0.706 (0.407)	0.696 (0.373)	0.691 (0.369)	0.716 (0.472)
Blood urea nitrogen (mmol/L)	14.1 (7.15)	14.0 (6.84)	14.0 (6.61)	14.1 (7.44)
Chloride (mmol/L)	103 (3.57)	103 (3.52)	103 (3.51)	103 (3.60)
Creatinine (mg/dL)	1.04 (0.743)	1.04 (0.713)	1.04 (0.686)	1.05 (0.769)
Glucose (mg/dL)	115 (59.6)	115 (59.5)	115 (59.6)	115 (59.5)
Hemoglobin (g/dL)	14.9 (1.59)	15.0 (1.56)	15.0 (1.54)	14.9 (1.62)
INR	1.05 (0.282)	1.05 (0.287)	1.05 (0.278)	1.06 (0.282)
Platelet (1000/µL)	229 (72.5)	231 (71.3)	233 (70.7)	228 (73.7)
Potassium (mEq/dL)	4.23 (0.441)	4.23 (0.439)	4.23 (0.438)	4.22 (0.444)
Total protein (g/dL)	7.55 (0.670)	7.55 (0.664)	7.55 (0.661)	7.55 (0.678)
Sodium (mEq/dL)	139 (3.11)	139 (3.09)	139 (3.08)	139 (3.14)
White blood cell count (1000/µL)	7.19 (2.56)	7.20 (2.54)	7.20 (2.54)	7.20 (2.62)

*Treated during 2000–2015. Does not include patients treated after 2015

### Model performance

We predicted cirrhosis development at 1-, 3-, and 5-years using a laboratory covariate time window of 2 and 4 years, respectively. The average, standard deviation, and 95% confidence intervals for AUROC over 30 random splits are summarized in Table 2. The misclassification results for all 6 combinations of outcome prediction windows and covariate time windows are shown in Table 3. To investigate the effect and significance of each predictor, we fit the 1-, 3- and 5-year outcome prediction model on the full cohort of data. The summary of model fitting is shown in Additional file 1: Tables S2–S4. The *p* values in the summary table reflect the significance of each variable’s longitudinal trajectory values in predicting cirrhosis. Cirrhosis predictors such as AST and platelets had extremely small *p* values (< 0.0001). SVR was highly significant in explaining cirrhosis outcomes.

**Table 2:** AUROC for cirrhosis prediction after 1-, 3-, and 5-years of follow up using 2- and 4-year laboratory data windows

	Follow-up interval		
	1-year	3-years	5-years
2-year laboratory window (n)	34,185	30,153	28,626
AUROC (95% CI)	0.815 (0.813–0.817)	0.811 (0.810–0.812)	0.794 (0.794–0.795)
4-year laboratory window (n)	25,953	20,915	18,198
AUROC (95% CI)	0.796 (0.794–0.798)	0.781 (0.779–0.783)	0.792 (0.791–0.793)

n represents the average sample size of test data for 30 splits

**Table 3:** Misclassification table

Follow-up interval, laboratory window (years)	Test sample (n)*	Event proportion	AUROC	Brier score	Best cut-off	Specificity	Sensitivity	PPV	NPV
1, 2	34,081	0.030	0.815	0.028	0.057	0.737	0.749	0.080	0.990
3, 2	30,117	0.161	0.811	0.133	0.059	0.767	0.719	0.373	0.934
5, 2	28,604	0.329	0.794	0.274	0.056	0.750	0.703	0.579	0.838
1, 4	25,934	0.026	0.796	0.031	0.105	0.731	0.712	0.066	0.990
3, 4	20,765	0.083	0.781	0.069	0.104	0.732	0.703	0.191	0.965
5, 4	18,164	0.230	0.792	0.172	0.105	0.723	0.721	0.438	0.897

*Sample size is based on the selected representative split

### Sensitivity analysis

The average AUROC using APRI > 2 gives 0.815 (95% CI 0.813–0.817) and using APRI > 1 gives 0.708 (95% CI 0.706–0.710) (for 1 year prediction model on 2-year lab accrual window evaluation).

## Discussion

A Cox model using time-varying covariates and a flexible time accrual window for longitudinal laboratory data achieved excellent discrimination for cirrhosis prediction at 1-, 3-, and 5-years among patients with HCV. Our study is the first to successfully use a large administrative dataset with a time-varying covariates model to predict future cirrhosis outcomes in HCV patients with and without SVR. This approach achieved high AUROCs for predicting the development of cirrhosis, as assessed by serial APRI score, and performed well at up to five years compared to previous models that were limited by fixed laboratory covariates and shorter follow up time [15].

We developed a novel approach to prediction by transforming longitudinal laboratory variables into time-varying covariates, allowing us to use each patient’s full spectrum of laboratory data instead of reducing the laboratory data to summary values. Unlike earlier models constructed exclusively for patients with viremic HCV, we included antiviral treatment as a time-varying covariate. Our model is therefore generalizable to both treated and viremic patients with HCV. All six combinations of laboratory data windows (2-or 4-years) and cirrhosis prediction windows (1-, 3-, or 5-years) produced excellent AUROCs. Taken together, our method accurately predicted risk of cirrhosis without inducing obvious bias due to the selection of the prediction window length.

Our study benefited from a very large HCV population drawn from the VHA healthcare network, which oversees the largest single cohort of patients with HCV in the US. We had access to comprehensive laboratory, demographic, and pharmacy data for all patients. VHA users tend to be older and more likely to be male than the general US population, so results should be extrapolated cautiously to other cohorts. Our conclusions are tempered by the use of a laboratory surrogate (two consecutive APRI scores > 2) to mark the development of cirrhosis rather than liver biopsy or transient elastography results, though prior studies have confirmed APRI as an excellent surrogate for biopsy-proven cirrhosis [17]. We selected this method due unknown validity of transient elastography values after HCV treatment, and the small proportion undergoing serial liver biopsy after antiviral therapy. In addition, we sought a surrogate cirrhosis endpoint that would be practical for others to replicate in administrative datasets and in resource-limited settings. Nevertheless, although APRI is considered a reliable laboratory marker of cirrhosis, a small amount of cirrhosis misclassification likely occurred. As a linear model, the time-varying covariates Cox model can only reflect a linear effect between the predictors and the outcome and therefore may not fully represent a non-linear relationship. We note that approximately 30% of the treated patients in our cohort received an interferon-based regimen due to the time period involved. Though such regimens are obsolete, there is no scientific reason to suspect that the type of regimen used would alter the risk of subsequent cirrhosis development after SVR or change the conclusions of the study. Finally, our data sources lacked results for laboratory testing or antiviral treatment conducted outside the VHA system. This model may not be generalized to non-Veteran populations and future external validation studies are needed to assess performance.

## Conclusions

Our model has many potential applications for predicting cirrhosis given the expanding population of patients with HCV now achieving SVR after antiviral treatment. For example, as more HCV patients successfully achieve SVR, practitioners will need tools to identify those at continued risk for cirrhosis despite antiviral therapy. Incorporating predictive models into HCV registries or other population-based systems may serve to identify patients who require continued specialty care and disease monitoring after HCV eradication. Furthermore, health care systems could also use cirrhosis prediction tools to estimate and prepare for the future burden of disease among persons with HCV, with and without treatment. Our novel time-varying covariates Cox model provides an accurate method for predicting cirrhosis that improves upon earlier models and can be applied at scale in large administrative datasets using widely available laboratory markers.

## Supplementary Information

**Additional file 1: **Supplementary Material.

## Data Availability

These analyses were performed using data from the Corporate Warehouse Domains that are available only within the US Department of Veterans Affairs firewall in a secure research environment, the VA Informatics and Computing Infrastructure (VINCI). In order to comply with VA privacy and data security policies and regulatory constraints, only aggregate summary statistics and results of our analyses are permitted to be removed from the data warehouse for publication. The authors have provided detailed results of the analyses in the paper. These restrictions are in place in order to maintain Veteran privacy and confidentiality. Access to these data can be granted to persons who are not employees of the VA; however, there is an official protocol that must be followed for doing so. The authors also confirm that VA policies are currently being developed that should allow an interested researcher to obtain a de-identified, raw dataset upon request with a data use agreement. Those wishing to access the data that were used for this analysis may contact Jennifer Burns, MHSA, who is a senior data manager at the VA Center for Clinical Management Research, to discuss the details of the VA data access approval process. Her contact information is as follows: Email: Jennifer.Burns@va.gov UM North Campus Research Complex, Department of Veterans Affairs, 2800 Plymouth Road Bldg 16, Ann Arbor, MI.

## Abbreviations

HCV: Hepatitis C virus
SVR: Sustained virologic response
APRI: AST-to-platelet ratio index
ICD: International Classifications of Diseases
AST: Aspartate aminotransferase
ALT: Alanine aminotransferase
BMI: Body mass index
AUROC: Area under receiver operating curve
PPV: Positive predictive value
NPV: Negative predictive value
DAA: Direct acting anti viral
