# Supplementary material for: Adapted time-varying covariates Cox model for predicting future cirrhosis development performs well in a large hepatitis C cohort
Source: BMC Med Inform Decis Mak. 2021 Dec 14;21:347. doi: 10.1186/s12911-021-01711-7 (PMC8670121; doi:10.1186/s12911-021-01711-7)
Supplement: Supplementary file 1 — Additional file 1: Supplementary Material. [file 12911_2021_1711_MOESM1_ESM.docx]

Supplemental Table 1: ICD9 and ICD10 codes used for condition definitions

|  | ICD9 |  | ICD10 |
| --- | --- | --- | --- |
| **Hepatocellular carcinoma** | 155.0 | **Hepatocellular carcinoma** | C22.0 |
|  |  |  |  |
| **CIRRHOSIS** |  |  |  |
| Alcoholic Cirrhosis Of Liver | 571.2 | Alcoholic cirrhosis without ascites | K70.30 |
| Cirrhosis Of Liver Without Mention Of Alcohol | 571.5 | Other cirrhosis of liver | K74.69 |
|  |  | Unspecified cirrhosis of the liver | K74.60 |
|  |  | Pigmentary cirrhosis of the liver | E83.110 |
|  |  | Cirrhosis (of liver) with toxic liver disease | K71.7 |
| Esophageal Varices Without Mention Of Bleeding | 456.1 | Esophageal varices, no bleeding | I85.00 |
| Esophageal Varices In Diseases Classified Elsewhere, Without Mention Of Bleeding | 456.21 | Gastric Varices, no bleeding | I86.40 |
|  |  | Secondary esophageal varices, no bleeding | I85.10 |
| Esophageal Varices With Bleeding | 456.0 | Esophageal varices, with bleeding | I85.01 |
| Esophageal Varices In Diseases Classified Elsewhere, With Bleeding | 456.20 | Gastric Varices, with bleeding | I86.41 |
|  |  | Secondary esophageal varices, with bleeding | I85.11 |
| Ascites | 789.5 | Alcoholic cirrhosis with ascites | K70.31 |
| Other ascites | 789.60 | Ascites in alcoholic hepatitis | K70.11 |
|  |  | Ascites in toxic liver disease with chronic active hepatitis | K71.51 |
|  |  | Other ascites | R18.8 |
| Spontaneous bacterial peritonitis | 567.23 |  | K65.2 |
| Hepatic encephalopathy | 572.2 | Hepatic failure, unspecified with coma | K72.91 |
|  |  | Encephalopathy, unspecified | G93.40 |
|  |  | Chronic failure with coma | K72.11 |
|  |  | Hepatic failure with coma | K72.91 |
| Hepatorenal syndrome | 572.5 |  | K76.7 |
| Hepatopulmonary syndrome | 573.5 |  | K76.81 |

Supplemental Table 2: Summary of 1 year outcome prediction model on full cohort

|  | coefficient | hazard ratio | standard error (coeff) | z | p-value |
| --- | --- | --- | --- | --- | --- |
| Albumin | -0.5461 | 0.5792 | 0.0157 | -34.8 | <.001 |
| Alkaline phosphatase | 0.1582 | 1.1714 | 0.009 | 17.53 | <.001 |
| ALT | 0.0307 | 1.0312 | 0.004 | 7.652 | <.001 |
| AST | 0.5346 | 1.7068 | 0.0071 | 75.529 | <.001 |
| Total bilirubin | 0.0849 | 1.0886 | 0.0108 | 7.841 | <.001 |
| BUN | -0.0064 | 0.9936 | 0.0011 | -5.714 | <.001 |
| Chloride | -0.0041 | 0.9959 | 0.0023 | -1.757 | 0.08 |
| Creatinine | 0.0157 | 1.0158 | 0.0085 | 1.976 | 0.48 |
| Glucose | -0.0001 | 0.9999 | 0.0001 | 0.94 | 0.35 |
| Hemoglobin | -0.024 | 0.9763 | 0.0044 | -5.458 | <.001 |
| Platelets | -0.0116 | 0.9885 | 0.0001 | -83.162 | <.001 |
| Potassium | -0.0224 | 0.9778 | 0.0158 | -1.418 | 0.16 |
| Sodium | -0.0151 | 0.9850 | 0.0028 | -5.418 | <.001 |
| White blood cell count | 0.0179 | 1.0181 | 0.0031 | 5.879 | <.001 |
| APRI | -0.0043 | 0.9957 | 0.0069 | -0.618 | <.001 |
| AST: ALT ratio | 0.1069 | 1.1128 | 0.0069 | 15.594 | <.001 |
| SVR | -1.3737 | 0.2532 | 0.101 | -13.6 | <.001 |
| Age at first APRI | -0.0069 | 0.9931 | 0.0009 | -7.544 | <.001 |
| Hispanic race | 0.4054 | 1.4999 | 0.0301 | 13.446 | <.001 |
| Missing race | 0.3397 | 1.4045 | 0.029 | 11.729 | <.001 |
| Other race | 0.2522 | 1.2869 | 0.0507 | 4.973 | <.001 |
| White race | 0.3264 | 1.3860 | 0.0156 | 20.877 | <.001 |
| Male sex | 0.1145 | 1.1213 | 0.0447 | 2.561 | 0.01 |

Supplemental Table 3: Summary of 3 year outcome prediction model on full cohort

|  | coefficient | hazard ratio | standard error (coeff) | z | p-value |
| --- | --- | --- | --- | --- | --- |
| Albumin | -0.3906 | 0.6766 | 0.0198 | -19.777 | <.001 |
| Alkaline phosphatase | 0.2251 | 1.2524 | 0.0157 | 14.326 | <.001 |
| ALT | 0.0028 | 1.0028 | 0.0076 | 0.37 | 0.71 |
| AST | 0.4173 | 1.5178 | 0.0117 | 35.801 | <.001 |
| Total bilirubin | 0.0840 | 1.0877 | 0.0162 | 5.188 | <.001 |
| BUN | -0.0071 | 0.9929 | 0.0014 | -5.013 | <.001 |
| Chloride | 0.0016 | 1.0016 | 0.0029 | 0.565 | 0.57 |
| Creatinine | 0.0514 | 1.0528 | 0.0098 | 5.235 | <.001 |
| Glucose | 0.0000 | 1 | 0.0002 | 0.21 | 0.83 |
| Hemoglobin | -0.0143 | 0.9858 | 0.0055 | -2.611 | 0.009 |
| Platelets | -0.0080 | 0.9921 | 0.0002 | -48.789 | <.001 |
| Potassium | 0.0128 | 1.0129 | 0.0192 | 0.668 | 0.5 |
| Sodium | -0.0200 | 0.9802 | 0.0034 | -5.866 | <.001 |
| White blood cell count | 0.0195 | 1.0197 | 0.0037 | 5.302 | <.001 |
| APRI | 0.1031 | 1.1086 | 0.0123 | 8.4 | <.001 |
| AST: ALT ratio | 0.1022 | 1.1076 | 0.0082 | 12.486 | <.001 |
| SVR | -1.6790 | 0.1865 | 0.2046 | -8.205 | <.001 |
| Age at first APRI | -0.0007 | 0.9929 | 0.0011 | -6.244 | <.001 |
| Hispanic race | 0.3942 | 1.4832 | 0.0366 | 10.757 | <.001 |
| Missing race | 0.2445 | 1.277 | 0.0383 | 6.389 | <.001 |
| Other race | 0.2446 | 1.2771 | 0.0616 | 3.972 | <.001 |
| White race | 0.2875 | 1.3331 | 0.0186 | 15.431 | <.001 |
| Male sex | 0.1363 | 1.1461 | 0.0526 | 2.592 | 0.009 |

Supplemental Table 4: Summary of 5 year outcome prediction model on full cohort

|  | coefficient | hazard ratio | standard error (coeff) | z | p-value |
| --- | --- | --- | --- | --- | --- |
| Albumin | -0.3564 | 0.7002 | 0.0243 | -14.698 | <.001 |
| Alkaline phosphatase | 0.2046 | 1.2271 | 0.0226 | 9.04 | <.001 |
| ALT | -0.0053 | 0.9948 | 0.0105 | -0.499 | 0.61 |
| AST | 0.3691 | 1.4464 | 0.0163 | 22.595 | <.001 |
| Total bilirubin | 0.0556 | 1.0571 | 0.0230 | 2.419 | 0.015 |
| BUN | -0.0053 | 0.9948 | 0.0018 | -2.965 | 0.003 |
| Chloride | -0.0001 | 0.9999 | 0.0035 | -0.017 | 0.986 |
| Creatinine | 0.0514 | 1.0528 | 0.0126 | 4.075 | <.001 |
| Glucose | 0.0001 | 1.0001 | 0.0002 | 0.404 | 0.686 |
| Hemoglobin | -0.0006 | 0.9994 | 0.0067 | -0.095 | 0.924 |
| Platelets | -0.0063 | 0.9937 | 0.0002 | -32.721 | <.001 |
| Potassium | -0.0118 | 0.9883 | 0.0234 | -0.505 | 0.614 |
| Sodium | -0.0118 | 0.9882 | 0.0042 | -2.842 | <.001 |
| White blood cell count | 0.0245 | 1.0248 | 0.0044 | 5.571 | <.001 |
| APRI | 0.1137 | 1.1204 | 0.0183 | 6.213 | <.001 |
| AST: ALT ratio | 0.0944 | 1.099 | 0.0107 | 8.793 | <.001 |
| SVR | -1.8920 | 0.1508 | 0.3167 | -5.974 | <.001 |
| Age at first APRI | -0.0082 | 0.9918 | 0.0014 | -5.722 | <.001 |
| Hispanic race | 0.3357 | 1.3989 | 0.0451 | 7.442 | <.001 |
| Missing race | 0.2116 | 1.2356 | 0.0476 | 4.444 | <.001 |
| Other race | 0.2647 | 1.303 | 0.0732 | 3.614 | <.001 |
| White race | 0.2450 | 1.2776 | 0.0223 | 10.98 | <.001 |
| Male sex | 0.1569 | 1.1698 | 0.0628 | 2.499 | 0.012 |
